# 981. An Investigation into Possible Nosocomial Clusters and On-Treatment Resistance Patterns in Candidemia

**DOI:** 10.1093/ofid/ofab466.1175

**Published:** 2021-12-04

**Authors:** Serin Edwin Erayil, Robert Todd, Susan E Kline, Anna Selmecki

**Affiliations:** University of Minnesota Medical School, Minneapolis, Minnesota

## Abstract

**Background:**

Candidemia has significant clinical implications, due to high rates of mortality and increasing resistance to antifungal drugs. *Candida auris* shows person-to-person transmission and survival on fomites. We aimed to determine if similar hospital transmission of *Candida* species, other than *C. auris*, is occurring. We analyzed candidemia infections for species, geographical, and temporal clusters, and clonality. We also aimed to study resistance patterns in individual patients on antifungal treatment. Here we present our current data from December 2019 – March 2021.

**Methods:**

Patients with candidemia were identified with the help of the clinical microbiology lab serving an urban health system. Isolates were stored prospectively as glycerol stocks at -80 C. Data were collected by retrospective chart review and described in terms of frequency distributions and percentages. Patient locations within the hospital setting were traced by Infection Prevention. A cluster was defined as the same species being isolated from ≥ 2 patients in the same unit within a 90-day period. Genomic DNA was isolated and whole genome sequencing was performed. Genomic data were visualized using the Yeast Mapping Analysis Pipeline. The Minimum Inhibitory Concentration (MIC) was determined using broth microdilution.

**Results:**

105 patients were identified from six hospitals (Table 1). Seven clusters of candidemia were identified (Table 2). Genome sequencing supported that all isolates from an individual patient were genetically related. No clonality was observed for isolates from different patients, including those representing two of the seven clusters (Figure 1). Loss of heterozygosity was detected in isolates collected 15 minutes apart in the same patient, indicating distinct populations. MICs remained the same at 0.5 ug/ml over 6 days in Patients l and m (Table 1). For Patient n, MICs remained at 0.5-1 ug/ml Day 1-8, but increased to 4 ug/ml on Day 9. No copy number variation was observed in these isolates.

Table 1. Species and resistance patterns of candidemia

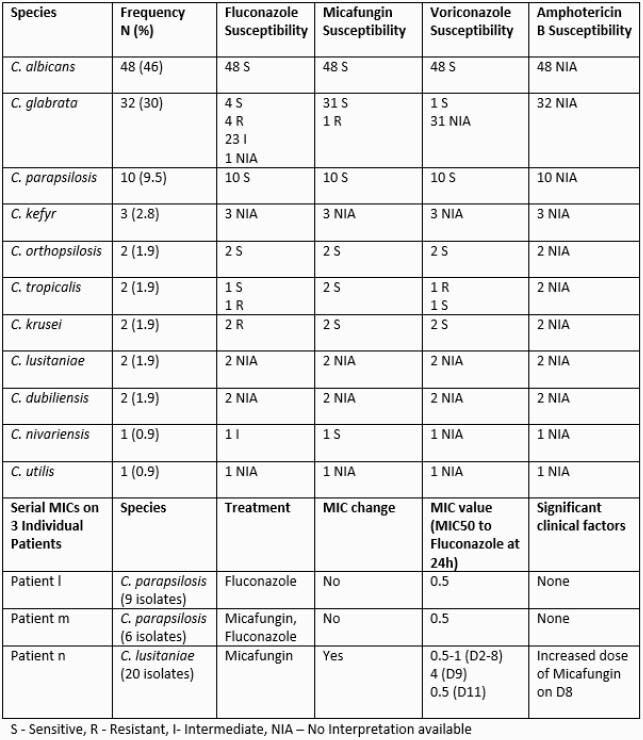

Table 2. Clusters of candidemia

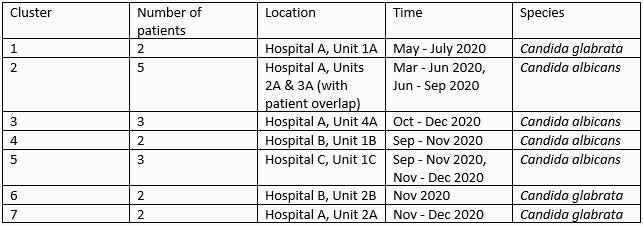

Figure 1. Yeast Mapping Analysis Pipeline for Clusters 3 and 4 – Candida albicans

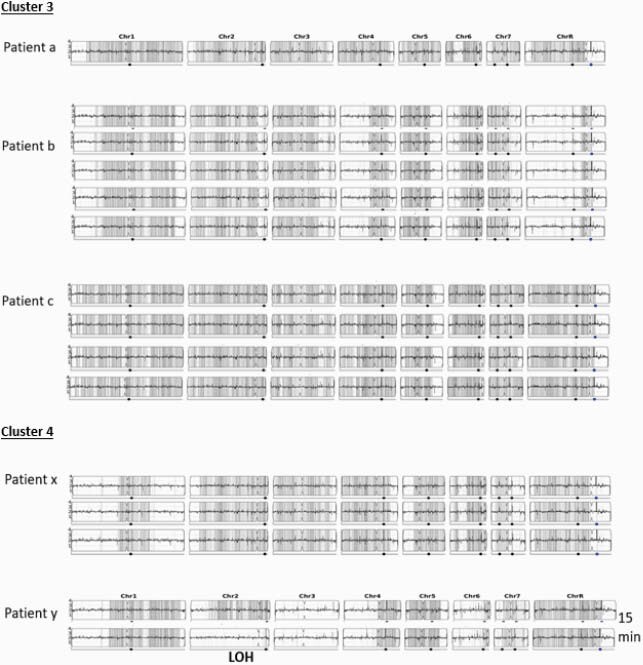

**Conclusion:**

We have not found evidence of hospital transmission of candida isolates in our investigations to date. We plan to evaluate clonality in the remaining 5 clusters. Future single nucleotide polymorphism analysis will determine if acquisition of point mutations is causing the increased MIC in Patient n.

**Disclosures:**

**All Authors**: No reported disclosures

